# Supplementary material for: The Glutathione Peroxidase Gene Family in Nitraria sibirica: Genome-Wide Identification, Classification, and Gene Expression Analysis under Stress Conditions
Source: Genes (Basel). 2023 Apr 21;14(4):950. doi: 10.3390/genes14040950 (PMC10137829; doi:10.3390/genes14040950)
Supplement: Supplementary file 1 [file genes-14-00950-s001.zip › Table S1.pdf]

Table S1. The sequences of *NsGPX* genes.

Gene	Sequence
NsGPX1	ATGGAAGAGGAAATGGACAGGAGTTCATTGAGGCATAGCAGCAACTGGGT<br>TTCTCTTCTTTTTATTGGATTTGCTCTCTTCTTGTATTTTTACAGATACC<br>CATTTTCTAATTCTTCTTCAAGTATGGCTGCCGAAGATGGTCCAAACTCC<br>ATTTACGACTTCACTGTTAAGGATATCCGAGGAAATGATATTAGTCTGAA<br>GGATTACAGTGGAAAGGTCCTCTTAATTGTGAATGTCGCTTCAAAATGCG<br>GTCTAACACAATCAAACTACAAGGAACTGAATGTCTTGTATGAGAAACAC<br>AAAGACCAAGGGTTTGAAATCTTGGCATTTCCTTGTAACCAGTTTGCTGG<br>ACAAGAGCCGGGAAACAAGGAGGAGATTCAGGAAACTGTCTGCACAATGT<br>TTAAAGCTGAATTCCCAGTTTTTGATAAGGTCGATGTGAATGGTAAGAAT<br>GCGGCGCCGCTCTACAAGTACCTAAAGTCACAGAAAGGAGGATTCTTAGG<br>AGATGCAATCAAGTGGAATTTCACAAAGTTTTTGGTTAACAAAGAAGGGA<br>AGGTTGTCGAGAGATATTCTCCCACCACGTCACCTCTTAAGATAGAGAAA<br>GATATACAGAATCTGTTGCAGTCGTCTTGA
NsGPX2	ATGGCGACCGCGATCCGACGGAGAATCAGCCTGAGGCTAACAAAAGAGAG<br>GTTGAGGAAGGTGTGGCTGAGGCAACGAAGGCGTTGGAGAGGAAAGAAAG<br>TGGAACTAAGGCAGAGGCAGCCCTTGGCGAAGAAATCTGGGCTTGCGAGA<br>AGCTTGGATCGGCTGGCGGAAGCGGTGACGAAGCAGGCTAGGGGCAGTCG<br>GACCAAGAGAGAGGCTTGGATGAAAAAACCCTTGCTTGATTTTCGATTTG<br>GAAACTGTATTTTGTGCAGGGGGAGTGGATTGACCACTTCAAATTACACT<br>GAACTTTCTCACCTATATGAGAAGTACAAGCCACAAGGATTTGAAATTCT<br>GGCTTTCCTATACAATCAGTTTGGGGGCAAGAGCTTGGAGCAAATCCAG<br>AGATCAAGCAATTTGCTTGTACCAGGTTTAAAGCAATTATAGTCCCATTT<br>CTTGTTGTTGACGTAAATGGACCAAACACCGCCCCAGTTTACCAGTGTTT<br>GAAGTCAAATGCAGGAGGATTTTTAGGGGATTTGATCAAGTGGAACTTTG<br>AGAAGTTCTTGGTTGACAAAAATGGTAAAGTTGTGGAGCGATATCCGCCA<br>ACAACATCTCCTCTCCAAATTGAGCTCACCAATCTTGGGCCGGATCCCAT<br>GGATCTCAGATTCACCATTTTTGGGCCGGATGCTCGTTTGAGCTTAGTGG<br>GCTCTGATACCATAGGTGCAGATTTTGAACTGATTGTGAACTTGATTGTT<br>CTCACAGCCGCTAGGGCTTGTACTACCATGGTGACACAGAAGGAGTTGTT<br>GCTTCAACAAATCATCTTGCTAAAATTTTATATTATTAATGAGTATGAGT<br>GA
NsGPX3	ATGCTCAGTTCATCAACTCGCCTTATCTTCAATCGAACTCTCAATATCAC<br>AGCTGTATCTGCTTCCGCATGTTTACCTCTAACGAAGAATTTATTTTCAG<br>ATTTTAAGAGATCGTTTTTGATATTTCCCCAATCGAAGCCGCTTTCATTT<br>CTTAATTCATCTAAGAAAGCAGAGGTTCCGAAGCCAGTTTTCTGCACTTT<br>GAGATCCGATCGTACCATGGCAAGCCAATCCAAGACATCAGTTCACGACT<br>TCACTGTCAAGGATGCTAGAGGAAATGATGTTGATCTTAGCATTTACAAG<br>GGGAAGCTCCTATTGATTGTCAATGTTGCTTCGCAATGTGGCCTGACCAA<br>CTCCAACTACACAGAGCTGAGTCAATTGTATGAGAAATACAAAAGTCAAG<br>GCTTGGAGATTCTAGCATTCCCGTGTAATCAGTTTGGAGCTCAAGAACCA<br>GGAAGTAATGAGCAGATTGTAGAATTTGCTTGCACTCGCTTTAAGGCTGA<br>GTATCCCATATTTGACAAGGTTGATGTGAATGGTGACAATGCTGCTCCAA<br>TATACAAGTTCCTGAAGTCCAGCAAAGGTGGGCTCTTTGGAGACAGTATC<br>AAGTGGAACTTCTCCAAGTTCTTGGTCGATAAAGATGGAAATGTTGTTGA<br>ACGTTATGCCCCTACCACATCACCTCTCAGCATCGAGGTCATGAAACTTG<br>GGAAGCTGATGATCGTGCCAAAGCATTATGAAATCGAGAGAACTAGAAAT<br>GTGTTCGGAGCGGTTTACATAGTTTTTAACTATCTAAACAACGGTTTCCG<br>TTTCTCTTCACTACTACTGGCTACCGTTGAGTGCTTCCGTCAACTGTACG

CTGCTCCTCCTCTTTCCTGCTATCTGCAGTTGCTTTGCTTGCCCTTCTCC
AAGCTTGGAAAAGTAGCGTATTACAAGATGACAAGCCAATCCGTGACGAA
TGCTGAATCAGTTTATGATTTCACTGTCAAGGATGCCAATGGCGATGATG
TAAATCTCAGCAAATATAGAGGGAAAGTGCTCCTCATAGTAAATGTTGCT
TCGAAATGTGGAATGACCAACTCCAACTACACAGAGTTGAATCACTTATA
CGAGAAGTACAAAGATCAAGGCTTGGAAATTCTGGCATTTCCATGCAATC
AATTTGGCGAAGAGGAGCCAGGAAGTAACGAAGAGATCAAAGAGCATGTC
TGCACACGCTTCAAATCAGAATTTCCCATCCTTGCCAAGATTGACGTGAA
CGGACAGGATGCTTCTCTGTTGTACAAGTTCTTAAAGTCCGGGAAATGGG
GCATTTTTGGGGATGATATTCAGTGGAACTTTGCCAAGTTCTTGGTTGAT
AAGAATGGTCAAGCCGTTGACCGTTATTACCCTACAACTTCCCCTCTTAG
CCTCGAGCGTGACATCGAGAAGCTGCTGAGCATGTCTTGCTGA

NsGPX4 ATGCTTAGTTCATCAACTCGCCTTATCTTCAATCGAACTCTCAATATCAC
AGCTGTATCTGCTTCCGCATGTTTACCTCTAACGAAGAATTTATTTTCAG
ATTCTAAGAGATCGTTTTTGATATTTCCCCAATCGAAGCCGCTTTCATTT
CTTAATTCATCTAAGAAAGCAGAGGTTCCGAAGCCAGTTTTCTGCACTTT
GAGATCCGATCGTACCATGGCAAGCCAATCCAAGACATCAGTTCACGACT
TCACTGTCAAGGATGCTAGAGGAAATGATGTTGATCTTAGCATTTACAAG
GGGAAGCTCCTATTGATTGTCAATGTTGCTTCGCAATGTGGCCTGACCAA
CTCCAACTACACAGAGCTGAGTCAATTGTATGAGAAATACAAAAGTCAAG
GCTTGGAGATTCTAGCATTCCCGTGTAATCAGTTTGGAGCTCAAGAACCA
GGAAGTAATGAGCAGATTGTAGAATTTGCTTGCACTCGCTTTAAGGCTGA
GTATCCCATATTTGACAAGGTTGATGTGAATGGTGACAATGCTGCTCCAA
TATACAAGTTCCTGAAGTCCAGCAAAGGTGGGCTCTTTGGAGACAGTATC
AAGTGGAACTTCTCCAAGTTCCTGGTCGATAAAGATGGAAATGTTGTTGA
ACGTTATGCCCCTACCACATCACCTCTCAGCATCGAGGTCATGAAACTTG
GGAAGTTGATGATCGTGCCAAAGCATTATGAAATCGAGAGAACTAGAAAT
GTGTTCGGAGCGGTTTACATAGTTTTTAACTATCTAAACAACGGTTTCCG
TTTCTCTTCACTACTACTGGCTACCGTTGAGTGCTTCCGTCAACTGTACG
CTGCTCCTCCTCTTTCCAGCTATCTGCAGTTGCTTTGCTTGCCCTTCTCC
AAGCTTGGAAAAGTAGCGTATTACAAGATGACAAGCCAATCCGTGACGAA
TGCTGAATCAGTTTATGATTTCACTGTCAAGGATGCCAATGGCGATGATG
TAAATCTCAGCAAATATAGAGGGAAAGTGCTCCTCATAGTAAATGTTGCT
TCGAAATGTGGAATGACCAACTCCAACTACACAGAGTTGAATCACTTATA
CGAGAAGTACAAAGATCAAGGCTTGGAAATTCTGGCATTTCCATGCAATC
AATTTGGCGAAGAGGAGCCAGGACGTAACGAAGAGATCAAAGAGCATGTC
TGCACACGCTTCAAATCAGAATTTCCCATCTTTGCCAAGATTGACGTGAA
CGGACAGGATGCTTCTCTGTTGTACAAGTTCTTAAAGTCCGGGAAATGGG
GCATTTTTGGGGATGATATTCAGTGGAACTTTGCCAAGTTCTTGGTTGAT
AAGAATGGGCAAGCCGTTGACCGTTATTACCCTACAACTTCCCCTCTTAG
CCTCGAGCGTGACATCGAGAAGCTGCTGAGCATGTCTTGCTGA

NsGPX5 ATGGTTTCGTCAGTCCCTTTTTCTGCAAATTTCTATTCACCTTTACACGC
TTTCCCACAGTCGAAAACCCATCCAGTTTCCTCCTCTTCTTCGTCTTTCT
CATGGCCTTCCATGGCTATTTTGACTCCTTCCATCAAATCCTCACTTGGG
TTTTCTAAATCAGGTTTTTTTCAACACGGGTTGTTTTTGCAGTCCTCAAT
TTTACCTGGGTTTTTCTTCAAAGCTCGCTCCTCGGGTGTTTATGCCACAG
CTGCTACTGAAAAGACCATCCATGACTACACTGTAAAGGATATTGATGGG
AAGGATGTTCCTCTTAGCAAATTTAAGGGGAAAGTTCTCTTGATTGTGAA
TGTTGCTTCAAAATGTGGATTGACCACTTCGAATTACACGGAACTCTCTC
ACTTATACGAGAAGTACAAGCCGCAAGGATTTGAAATTCTGGCTTTTCCA
TGCAATCAGTTTGGGGGGCAAGAGCCTGGAGCAAATCCAGAGATCAAGCA

	ATTTGCTTGTACCAGGTTTAAAGCAGAATTTCCCATTTTCGACAAGGTTG<br>ACGTAAATGGACCAAACACTGCCCCAGTTTACCAGTATTTAAAGTCAAAT<br>GCAGGAGGATTTTTAGGGGATTTGATCAAGTGGAACTTTGAGAAGTTCTT<br>GGTTGACAAAAATGGTAAAGTTGTGGAGCGATATCCGCCAACAACATCTC<br>CTCTGCAAATTGAGAAGGACATCCAGAAGCTTGTTACGGCATGA
NsGPX6	ATGGAAATGCCTGAGTCGAACAAAAGAGGGGCTGAGGAAGGTGTGGCTGA<br>GGCAGCGGAGGCGTCGAAGAGAAAAGATAGTGGAACTAAGGCAGAGGCAG<br>CCCTTGGCGAAGAAATCTCGATCTTTTCTTTGAATCCCAATGGATTGACC<br>ACTCCAAATTACAATGAACTTTCTCACTTATACGATAAGTACAAGCCGCA<br>AGGATTTGAAATTCTGGCTTTCCCATGCAATCAGTTTGGGGGGCAAGAGC<br>CTGGAGCAAATCTAGAGATCAAGCCATTTGCTTGTACCAAGTTTAAAGCA<br>GAATTCCCCATTTTCGACAAGTGGAACTTTGAAAAGTTCTTGGTTGACAA<br>AAATGGTAAAGTTGTGGAGCGCTATCCGCCAACAACATCTGCTCGCCAAA<br>TTGAGAAGGACATCCAGAAGGTTGTTGTGGCATAA
NsGPX7	ATGACTGATTTGGGTTTTGGCGATAATTACGAGCAGGATAGCAAGGGCAA<br>GGAAGTGGACCTCAGCATCTACAAAGGGAAGGTCTTACTCGTGGTCAACG<br>TCGCCTCCAAATGCGGGTTTACCGATTCAAATTACACGCAGTTGACTGAG<br>CTTTACAGCAAATACAAGGACAAAGGTTTTGAGGTTTTCGCATTTCCATG<br>CAACCAATTTCTAAACCAGGAGCCTGGGACGAGCCAGGAGGCGCAAGATT<br>TTGCATGTACCAGATACAAGGCTGAATATCCAGTTTTTCAAAAGGTTCGT<br>GTAAATGGTCCAAACACAGCACCCGTGTACCAATTCCTTAAAGCAAGTAA<br>ATCCACATTCTGGGGTCCAAGAATAAAGTGGAACTTCACCAAGTTCTTGG<br>TTGATAAAGAAGGCCATGTCATCGCTCGCTATGGCACTTCCACCGCTCCA<br>TTGGCTATCGAGGTTCACTTTTGCACCATCTCTCTCTCTAACACACATAC<br>TTGTGCACGCGCACACACTTTTTCTGATGACACTTTGTTCATTGTCTCAT<br>TGATCTCTAGTTAG
