# Supplementary material for: The Glutathione Peroxidase Gene Family in Nitraria sibirica: Genome-Wide Identification, Classification, and Gene Expression Analysis under Stress Conditions
Source: Genes (Basel). 2023 Apr 21;14(4):950. doi: 10.3390/genes14040950 (PMC10137829; doi:10.3390/genes14040950)
Supplement: Supplementary file 1 [file genes-14-00950-s001.zip › Table S2.pdf]

Table S2. qRT-PCR primers used to quantify *NsGPX* gene expression.

Gene		qRT-PCR Primers (5′-3′)	Tm (℃)
NsHis	F	AGGAGGCGTCGAGATTGGCGAGGTA	56.5
	R	AGGAGGCGTCGAGATTGGCGAGGTA	55.67
NsActin	F	TCGTGTTGCCCCTGAAGAACACCCCGT	58.8
	R	TGGATGGCGACGTACATAGCGGGCA	57.3
NsGPX1	F	TGGAAGAGGAAATGGACAGGAG	57.5
	R	TGGAGTTTGGACCATCTTCGG	57.3
NsGPX3	F	AATTTGGCGAAGAGGAGCCA	57.1
	R	GCCCCATTTCCCGGACTTTA	57.9
NsGPX4	F	AATTTGGCGAAGAGGAGCCA	57.1
	R	GCCCCATTTCCCGGACTTTA	57.9
NsGPX5	F	GTAAATGGACCAAACACTGCCC	56.7
	R	GAGGAGATGTTGTTGGCGGA	57.7
NsGPX7	F	GCCTCCAAATGCGGGTTTAC	57.3
	R	GCAAAATCTTGCGCCTCCTG	57.8
