# Supplementary material for: The Glutathione Peroxidase Gene Family in Nitraria sibirica: Genome-Wide Identification, Classification, and Gene Expression Analysis under Stress Conditions
Source: Genes (Basel). 2023 Apr 21;14(4):950. doi: 10.3390/genes14040950 (PMC10137829; doi:10.3390/genes14040950)
Supplement: Supplementary file 1 [file genes-14-00950-s001.zip › Table S3.pdf]

Table S3. Prediction of subcellular localization of NsGPX proteins

Protein_ID	Localizations	Signals	Cytoplasm	Nucleus	Extracellular	Cell membrane	Mitochondrion	Plastid	Endoplasmic reticulum	Lysosome/Vacuole	Golgi apparatus	Peroxisome
NsGPX1	Endoplasmic reticulum	Signal peptide|Transmembrane domain	0.3091	0.2121	0.2244	0.1993	0.1917	0.0873	0.6979	0.5162	0.6347	0.1366
NsGPX2	Extracellular	Signal peptide	0.3598	0.3233	0.5643	0.146	0.2196	0.2626	0.2246	0.2219	0.1586	0.3135
NsGPX3	Mitochondrion|Plastid	Mitochondrial transit peptide	0.2848	0.2494	0.1079	0.0344	0.7851	0.7219	0.1356	0.0619	0.1038	0.1367
NsGPX4	Mitochondrion|Plastid	Mitochondrial transit peptide	0.2747	0.2375	0.1071	0.0325	0.7903	0.7493	0.1209	0.0574	0.0967	0.1342
NsGPX5	Plastid	Chloroplast transit peptide	0.1432	0.1251	0.0589	0.0587	0.259	0.9903	0.0682	0.0541	0.0758	0.1831
NsGPX6	Nucleus	Nuclear localization signal	0.4126	0.7549	0.4492	0.1005	0.0414	0.0088	0.0614	0.0327	0.0333	0.0069
NsGPX7	Cytoplasm		0.5945	0.2957	0.3049	0.2812	0.4374	0.1226	0.2692	0.2209	0.2085	0.2358
