# Supplementary material for: The Glutathione Peroxidase Gene Family in Nitraria sibirica: Genome-Wide Identification, Classification, and Gene Expression Analysis under Stress Conditions
Source: Genes (Basel). 2023 Apr 21;14(4):950. doi: 10.3390/genes14040950 (PMC10137829; doi:10.3390/genes14040950)
Supplement: Supplementary file 1 [file genes-14-00950-s001.zip › Table S4.pdf]

Table S4. Kinds and numbers of known *cis*-elements in the promoter regions of *NsGPX* genes

	**Environmental Stress**			**Plant Hormones**				
	**MYB**	**MBS**	**light responsive element**	**SA**	**MeJA**	**GA**	**ABA**	**Aux**
NsGPX1	✓	✓	✓	✓	✓	✓	✓	
NsGPX2	✓							✓
NsGPX3	✓	✓	✓	✓	✓	✓	✓	
NsGPX4	✓	✓	✓	✓	✓	✓	✓	
NsGPX5	✓				✓			✓
NsGPX6	✓	✓	✓					
NsGPX7	✓							
