# Supplementary figures and images for: The Glutathione Peroxidase Gene Family in Nitraria sibirica: Genome-Wide Identification, Classification, and Gene Expression Analysis under Stress Conditions
Source: Genes (Basel). 2023 Apr 21;14(4):950. doi: 10.3390/genes14040950 (PMC10137829; doi:10.3390/genes14040950)

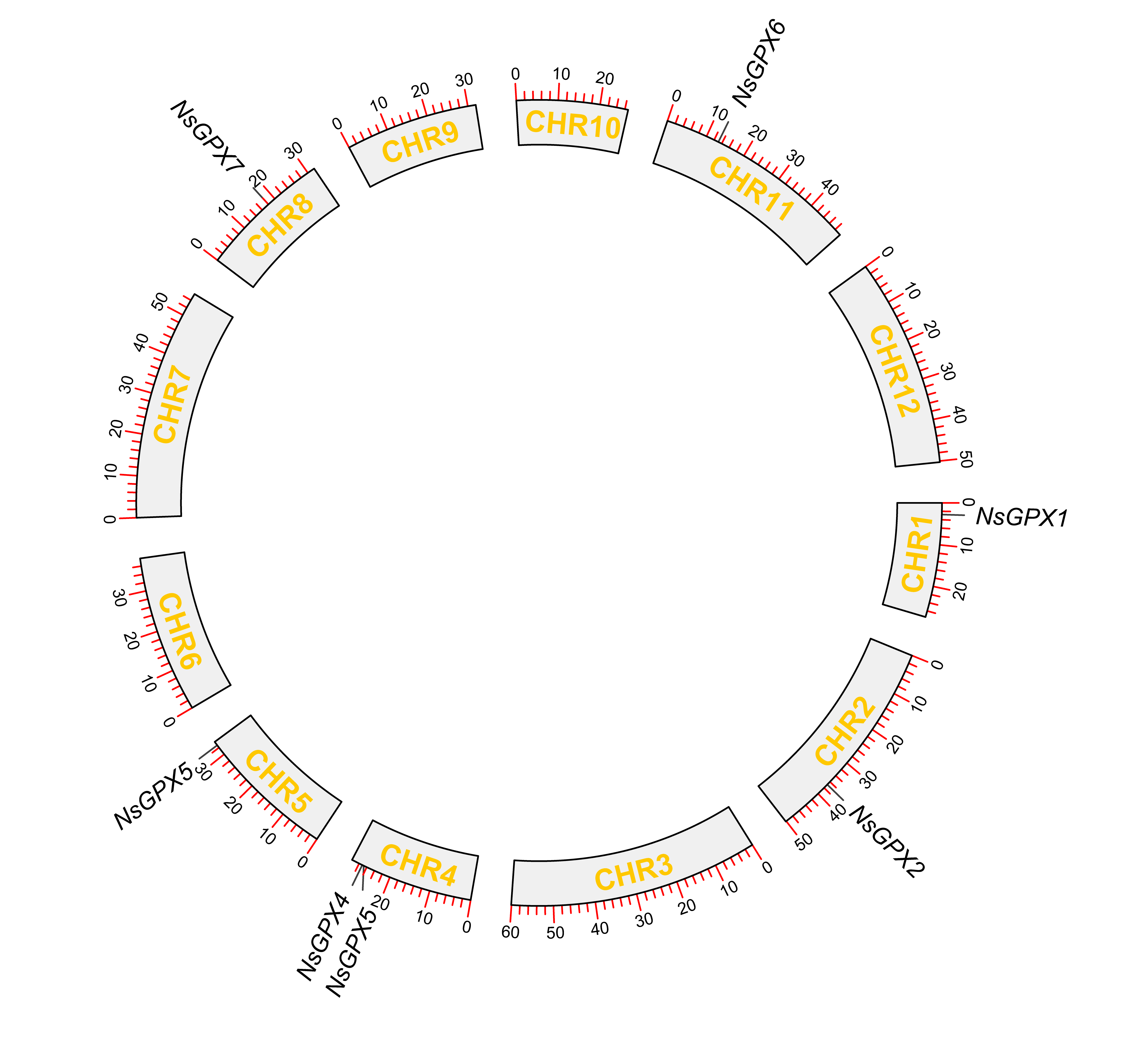

Supplement: Supplementary file 1 [file genes-14-00950-s001.zip › Figure S3.tif]
